# Mifepristone Reduces Insulin Resistance in Patient Volunteers with Adrenal Incidentalomas That Secrete Low Levels of Cortisol: A Pilot Study

**DOI:** 10.1371/journal.pone.0060984

**Published:** 2013-04-05

**Authors:** Miguel Debono, Rita Chadarevian, Richard Eastell, Richard J. Ross, John Newell-Price

**Affiliations:** 1 Academic Unit of Diabetes, Endocrinology and Reproduction, Department of Human Metabolism, University of Sheffield, Sheffield, United Kingdom; 2 Academic Unit of Bone Metabolism, Department of Human Metabolism, University of Sheffield, Sheffield, United Kingdom; 3 HRA Pharma, Paris, France; University of Warwick – Medical School, United Kingdom

## Abstract

**Background:**

Incidental adrenal masses are commonly detected during imaging for other pathologies. 10% of the elderly population has an ‘adrenal incidentaloma’, up to 20% of these show low-grade autonomous cortisol secretion and 60% of patients with autonomous cortisol secretion have insulin resistance. Cortisol excess is known to cause insulin resistance, an independent cardiovascular risk marker, however in patients with adrenal incidentalomas it is unknown whether their insulin resistance is secondary to the excess cortisol and therefore potentially reversible. In a proof of concept study we examined the short-term effects of glucocorticoid receptor (GR) antagonism in patients with an adrenal incidentaloma to determine whether their insulin resistance was reversible.

**Methodology/Principal Findings:**

In a prospective open-label pilot study, six individuals with adrenal incidentalomas and autonomous cortisol secretion were treated with mifepristone (a GR antagonist) 200 mg twice daily and studied for 4 weeks on a Clinical Research Facility. Insulin resistance at four weeks was assessed by insulin resistance indices, lnHOMA-IR and lnMatsuda, and AUC insulin during a 2-hour glucose tolerance test. Biochemical evidence of GR blockade was shown in all individuals and across the group there was a significant reduction in insulin resistance: lnHOMA-IR (1.0vs0.6; p = 0.03), lnHOMA-%beta (4.8vs4.3; p = 0.03) and lnMatsuda (1.2vs1.6; p = 0.03). Five out of six individuals showed a reduction in insulin AUC >7237 pmol/l.min, and in two patients this showed a clinically significant cardiovascular benefit (as defined by the Helsinki heart study).

**Conclusions:**

Short-term GR antagonism is sufficient to reduce insulin resistance in some individuals with adrenal incidentalomas and mild cortisol excess. Further assessment is required to assess if the responses may be used to stratify therapy as adrenal incidentalomas may be a common remediable cause of increased cardiovascular risk.

**Trial Registration:**

ClinicalTrials.gov NCT00721201

## Introduction

Adrenal masses incidentally disclosed on computed tomography (CT) scans, ‘adrenal incidentalomas’ are common. Prevalence increases during life such that they are found in ten percent of the population aged 70 years or more [1], [2]. Between five to twenty percent of these are associated with mild cortisol excess without the classical external features typically associated with Cushing's syndrome [3], often termed ‘subclinical Cushing's syndrome’, or ‘subclinical hypercortisolism’ [4]. One of cortisol's major functions is the regulation of glucose metabolism: it promotes gluconeogenesis by activation of phosphoenolpyruvate carboxykinase and glucose-6-phosphatase in the liver [5], [6]; inhibits insulin release in the pancreas [7]; decreases insulin sensitivity in skeletal muscle and adipose tissue [8]. Consequently, an important complication of cortisol excess is insulin resistance (IR) [9], a major independent marker of cardiovascular risk [10], and this is reflected by the fact that over 60% of individuals with adrenal incidentalomas and low grade cortisol secretion have IR, impaired glucose tolerance or diabetes mellitus compared to age, sex and BMI-matched controls [11]. However, as IR is common in the general population it is unknown whether the IR in patients with adrenal incidentalomas is secondary to the low level cortisol secretion or other factors.

The GR antagonist mifepristone (11-[4-(Dimethylamino)phenyl]-17-hydroxy-17-[1-propynyl]-[11ß,17ß]-estra-4,9-dien-3-one) has been shown to improve glucose tolerance in patients with overt Cushing's syndrome [12]. We hypothesized that insulin resistance in individuals with adrenal incidentalomas and low-grade cortisol excess without the classical external features typically associated with Cushing's syndrome might also be improved by short-term GR blockade if in these individuals IR is driven by cortisol excess. Our findings showed, for the first time, that even short-term GR blockade in these individuals improved insulin resistance. These data could inform the design of a much awaited prospective interventional randomized controlled study aimed to identify whether such an approach could be used as a prediction tool to stratify an affected individual to surgery, medical therapy or observation [13].

## Methods

### Ethics Statement & Participants

The study protocol was approved by the North Sheffield Research Ethics committee, and the Medicines and Health Regulatory Authority, UK and was performed according to the requirements of the Declaration of Helsinki as revised in 2000. Written informed consent was obtained from all participants. The study is reported according to the Transparent Reporting of Evaluations with Nonrandomized Designs (TREND) [14]. These are guidelines for the reporting of nonrandomised trials to help improve the clarity of a report and to encourage more detailed description of the study design and the findings. The statement presents a checklist for investigators to follow and which are mainly directed towards intervention evaluation studies. The protocol for this trial and supporting TREND checklist are available as supporting information; see Checklist S1 and Protocol S1.

This prospective open-label pilot study was performed at the Clinical Research Facility, Sheffield Teaching Hospitals NHS Foundation Trust and The University of Sheffield, UK between January 2010 and March 2010 in six patients. Patients were identified by continuous sampling from referrals to the Endocrine Investigation Unit as part of an incidentaloma work-up protocol at the Royal Hallamshire Hospital, Sheffield. It was planned to perform the study over 8 weeks, but we analyzed at four weeks, focusing on IR. Metabolic outcome was achieved and all 6 patients had IR indices and BP measurements suitable for analysis. After ingestion of 200 mg of mifepristone, peak concentrations are reached within 1 to 2 hours, there is an initial redistribution phase of 6–10 h followed by a plateau for 24 h or more. The terminal half-life (t_1/2_) is 30 hours. Dose-dependent antiglucocorticoid effects last for at least 24 hours after a single dose, hence significant effects would already be expected by a week [15], [16]. Two patients had been withdrawn after four weeks. All subjects were known to have an adrenal incidentaloma, with benign characteristics as assessed on CT, lacking external clinical features classically associated with Cushing's [4], [17], [18], but having evidence of excess cortisol as shown by lack of suppression of serum cortisol to <50 nmol/L (1.8 ug/dL) on 1 mg over-night dexamethasone suppression and 2 mg/day 48 hour low-dose dexamethasone suppression testing [18]. All were on stable anti-hypertensive treatment for at least three months and none were on anti-diabetes medications. Although this was an exploratory human subject study to assess if insulin resistance could be reversed and not a formal outcome study *per se*, it was still registered at Clinical trials.gov: ID NCT00721201.

### Study Design & End-points

**Figure 1:**
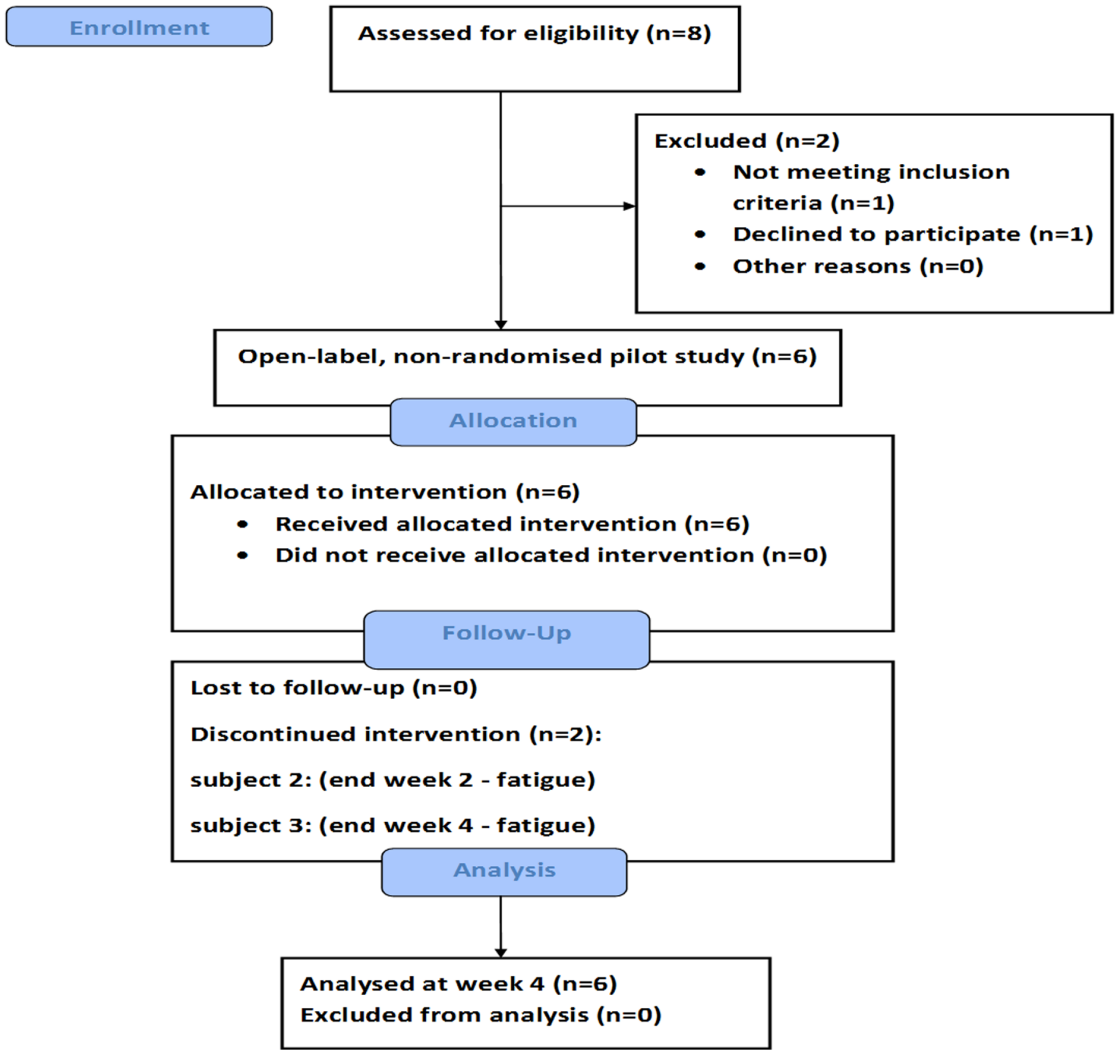
Flow chart indicating the different phases of the study to week four as recommended by the TREND statement [14]. Analysis performed at week four as all six patients had insulin resistance and blood pressure measurements suitable for analysis.

**Figure 2:**
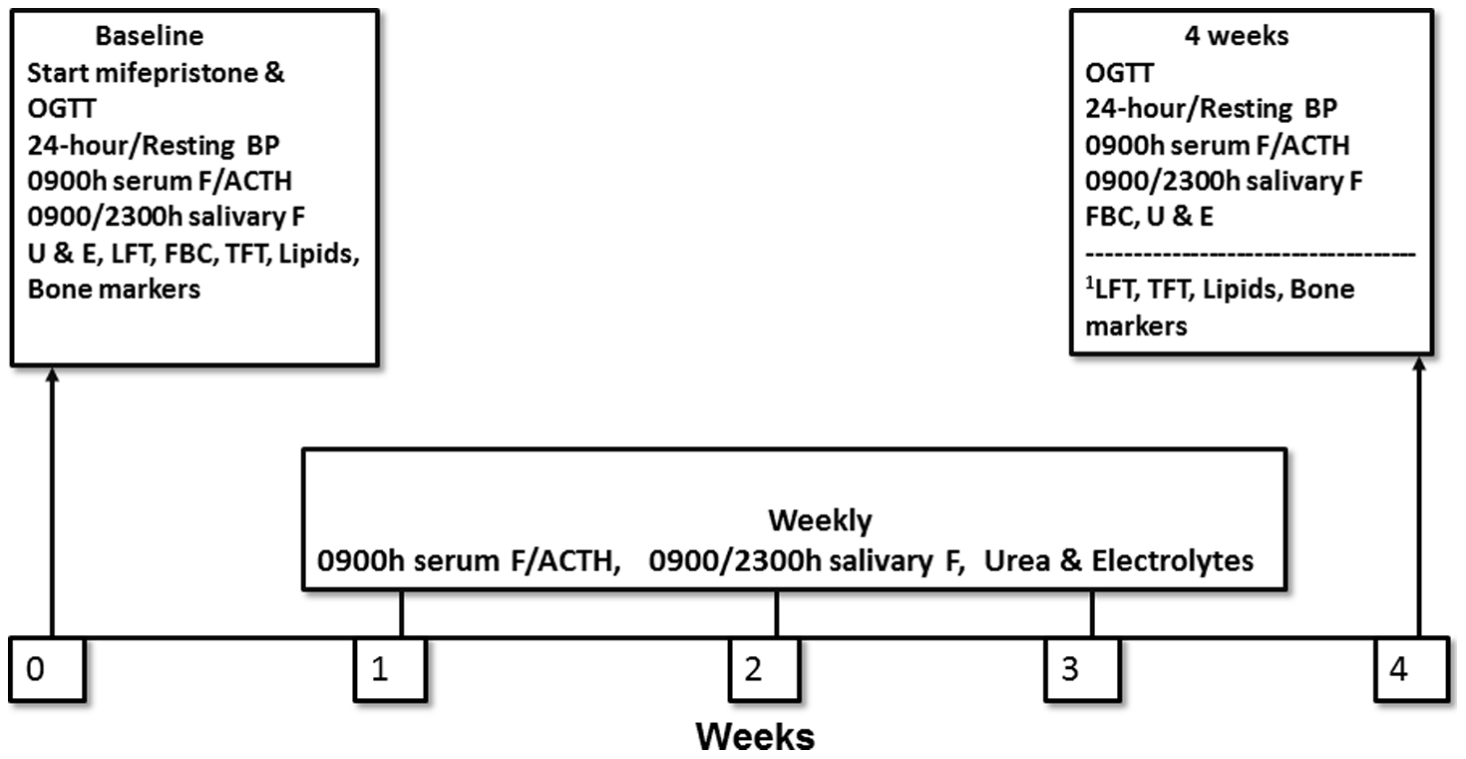
Study Design showing investigations and intervention performed on six patients from baseline to four weeks. One subject was withdrawn after end of second week. ^1^variables measured at baseline and end of study. OGTT: Oral Glucose Tolerance Test - insulin & glucose at 0, 15, 30, 60, 90, 120 min, F: cortisol, ACTH: Adrenocorticotropic Hormone, U & E: urea, electrolytes and creatinine, LFT: Liver Function Tests, FBC: Full Blood Count, TFT: Thyroid Function Tests.

The flow chart in Figure 1, as recommended by the TREND statement, describes the different phases and design of the study. One patient who was approached and screened did not meet the entry criteria, whilst one other declined to participate. Study visits occurred weekly up to week 4 and procedures at each visit are shown in Figure 2. The baseline glucose tolerance test was performed on the first day of treatment (treatment start) before the first dose of mifepristone whilst the baseline 24-hour ambulatory BP monitoring was performed between the first visit (screening) and second visit (treatment start). During each visit renal function, 0900 h and 2300 h salivary cortisol (representing free cortisol) and 0900 h plasma ACTH and serum cortisol (total cortisol) levels were assessed to confirm compliance and efficacy of GR blockade. All interventions were performed by the clinical research team on the Facility. Since mifepristone blocks the GR the expectation was a rise in plasma ACTH and serum and salivary cortisol by activation of the hypothalamo-pituitary-adrenal (HPA) axis, but with blockade of GR-mediated activity. Saliva samples were obtained using the Salivette tubes (cotton swab with citric acid preparation; Sarstedt, Numbrecht). Subjects were instructed to refrain from brushing their teeth, smoking, eating or drinking anything for at least 60 min prior to sampling. To provide a saliva sample, patients were asked to chew on the cotton swab for 1–2 min and then place it back in the plastic container. The bone formation marker serum osteocalcin was measured in a 0900 h fasting sample, and the second morning urine sample was used to measure the bone resorption marker urine N-telopeptide crosslinks of type 1 collagen (NTX). Full clinical assessment including resting BP, weight and temperature was made at each visit - monitoring for symptoms and signs for adrenal insufficiency can only be performed on a clinical, and not biochemical, basis when on mifepristone. The oral dose of mifepristone was kept the same at 200 mg twice a day at 0900 h and 2100 h, one hour before any food, throughout the study. Although once daily dosing might have sufficed, twice daily dosing was chosen in an effort to maintain as constant a GR blockade effect as possible.

To establish a normal 95% reference range for IR we analyzed data from the Helsinki Policemen study, a 22 year follow up investigating what level of IR, as reflected by insulin AUC on an OGTT, predicted cardiovascular events. By taking the mean±2SD of the values of those followed for 22 years (n = 755) and in whom no cardiovascular event was observed, we define the upper end of the 95% reference range of insulin AUC associated with no cardiovascular risk as 63420 pmol/l.min (26940±2SD pmol/l.min) [10]. In addition we took values <26940 pmol/l.min to represent definite normalization of AUC and only a decrease in insulin AUC greater than 7237 pmol/l.min, during a 2-hour OGTT, was considered significant as this exceeds biological variation [19]. The insulin AUC was appropriate for our group as in patients with fasting insulin levels less than 200 pmol/l the insulin AUC is reproducible [19]. We chose insulin AUC on OGTT as our primary estimate of IR as this allowed direct comparison to the seminal Helsinki heart study data examining the long term effects of IR on cardiovascular risk. Resting and 24-hour ambulatory blood pressure as measured using standard British Hypertension Society approved monitors were other primary end points.

Secondary end points were the means for fasting glucose, fasting insulin, the homeostasis model assessment of IR and beta cell function (HOMA-IR and HOMA-%beta) [20], and the Insulin Sensitivity Index (ISI) – Matsuda [21], as calculated from an oral glucose tolerance test. The difference in fasting lipids and bone turnover markers at study end were also measured.

### Assays

Total serum cortisol was measured in a Siemens Advia Centaur Cortisol assay: analytical range 5.5–2069 nmol/l; inter-assay co-efficient of variation (CV), 6.2% at 134 nmol/l, 5.5% at 491 nmol/l and 6.0% at 836 nmol/l. Plasma ACTH was measured in a Siemens Immulite 2000 chemiluminescent assay: Analytical range 1.1–275 pmol/l; inter-assay CVs 6.4% at 7 pmol/l, 6.5% at 105 pmol/l. Salivary cortisol was measured by Liquid Chromatography Tandem Mass spectrometry (LC-MS/MS) as previously described [22]. Glucose was measured using the Hexokinase method, Beckman Coulter Inc, with CV 2.6% at 3.3 mmol/l and 2.1% at 20.3 mmol/l. Insulin was measured by Siemens Advia Centaur chemiluminescent immunometric assay with a CV of 5.3% at 222 pmol/l, 4.9% at 760.5 pmol/l and 5.8% at 1409 pmol/l. The analysis of serum osteocalcin and urine NTX was performed by Cobas e411 Autoanalyser (Roche Diagnostics) and the Ortho Clinical Vitros device respectively, with interassay CV's of 4.5% and 6.6% respectively. Urine NTX was expressed as a ratio to urinary creatinine. Standard biochemistry and hematological methods were used for renal, liver and thyroid function, lipid profiles and the full blood count.

### Statistical Analysis

Statistical analyses were performed using SPSS version 16 (SPSS, Chicago, IL, USA) and Microsoft Office Excel 2010. Rates were calculated for categorical data, and means and 95% confidence intervals for continuous data. We used non-parametric Wilcoxon's signed rank test for repeated measures to analyze the effect of up to four weeks of mifepristone treatment on variables of glucose, insulin and insulin sensitivity, resting and 24 hour BP, bone markers, lipids, renal, liver and thyroid function. As the distribution of fasting insulin levels is usually skewed measures of insulin sensitivity were log-transformed [23]. As per protocol, any missing data from premature withdrawal was replaced by at least one post-baseline measured efficacy parameter that was carried forward. Efficacy evaluation was based on the intention to treat population (ITT), where all patients who received at least one dose of mifepristone and from whom at least one efficacy measurement is obtained after the study treatment start were analysed. All significances were two-sided and values of p<0.05 were regarded as statistically significant.

## Results

### Baseline Characteristics

Demographic data for each subject and means (±95%CI) are shown in Table 1. Baseline characteristics were similar to the target population of interest [24], [25].

**Table 1:** Demographic data – Subject Baseline Characteristics.

Subject	1	2	3	4	5	6	Mean (±95% CI)
Gender	M	F	F	F	M	M	
Age (yrs) (range)	72	58	74	56	64	75	67 (58–75)
Mean Weight (kg)	86.0	75.6	70.9	109.0	101.5	110.0	92.2 (74.3–110.0)
Mean BMI (kgm^2^)	29.1	31.1	24.5	43.7	32.0	39.9	33.4 (26.9–40.5)
Serum Cortisol post ONDST (nmol/l)	67.9	104.0	83.9	88.8	59.9	73.9	79.7 (63.2–96.3)
Serum Cortisol post LDDST (nmol/l)	80.0	54.1	85.0	105.1	80.0	61.0	77.5 (58.2–96.8)
0900 h salivary cortisol (nmol/l)	4.7	6.8	5.2	3.4	5.3	3.4	4.8 (3.4–6.2)
2300 h salivary cortisol (nmol/l)	0.5	0.6	0.9	3.0	2.1	0.5	1.3 (0.2–2.4)
ACTH (pmol/l)	1.9	1.1	2.0	4.1	1.1	1.2	1.9 (0.9–2.9)
Side of adenoma	R	R	L	R	R	R	
Size of adenoma (cm)	3.8	2.5	3.6	3.5	2.7	2.0	3.02 (2.3–3.8)
24-hour blood pressure	136/91	143/75	149/86	150/86	138/81	147/77	
Fasting glucose (mmol/l)	4.8	6.3	4.7	4.9	5.1	6.5	5.4 (4.6–6.2)
Fasting insulin (pmol/l)	44.4	124.2	34.0	177.1	69.2	86.8	76.4 (39.9–146.4)^1^

ONDST: Overnight dexamethasone suppression test; LDDST: 48-hour 2 mg low dose dexamethasone suppression test; R = Right, L = Left;

1 Geometric mean.

### Hypothalamo-pituitary-adrenal axis analysis

**Figure 3:**
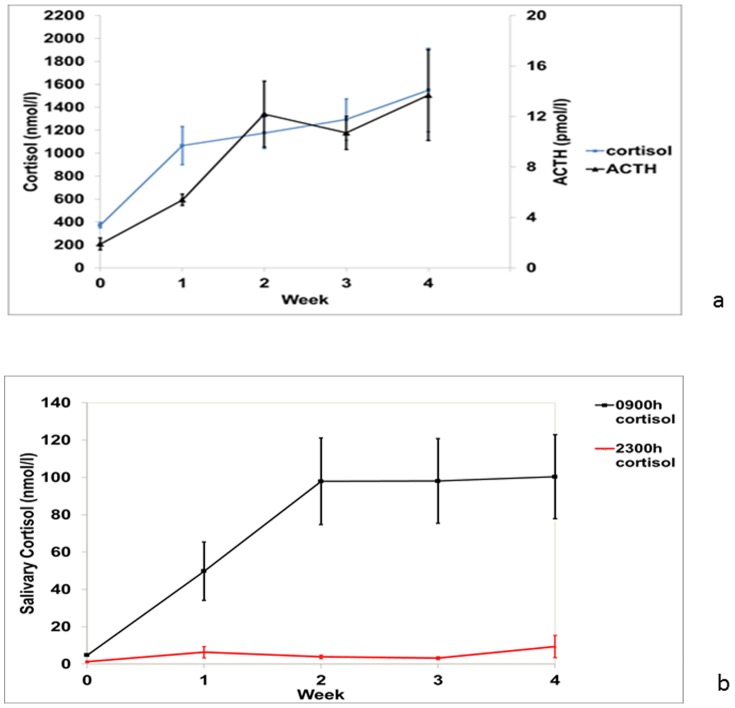
a: Graph showing mean ±SEM 0900 h serum cortisol and ACTH levels at baseline and 4 weeks. Activation of the HPA axis, that is a rise in serum cortisol and ACTH, is already evident one week after starting mifepristone. **b: Graph showing mean ±SEM 0900 h and 2300 h salivary cortisol levels at baseline and 4 weeks.** Circadian rhythm of cortisol is maintained evidenced by high levels at 0900 h and low levels at 2300 h, but amplified by the use of mifepristone 200 mg twice per day.

Mifepristone blocked glucocorticoid action as evidenced by activation of the HPA axis. Baseline mean serum cortisol level was 372.5 nmol/l (95%CI 309.0–435.9 nmol/l), this increased progressively in each subject, such that at one week levels had increased significantly (1065 nmol/l 95% CI 636.0–1495.4 nmol/l; p = 0.03) and further increased by 4 weeks (1551 nmol/l (95%CI 615–2486 nmol/l; p = 0.03). Baseline mean plasma ACTH was low at 1.9 pmol/l (95%CI 0.7–3.1 pmol/l) whilst plasma ACTH levels at 4 weeks were 13.6 pmol/l (95%CI 4.4–22.9 pmol/l; p = 0.03) (Figure 3a). Baseline mean salivary cortisol levels at 0900 h and 2300 h were 4.8 nmol/l (95%CI 3.4–6.2 nmol/l) and 1.3 nmol/l (95%CI 0.2–2.4 nmol/l), respectively. There was a significant increase in salivary cortisol over 4 weeks at 0900 h (99.3 nmol/l; p = 0.03) and 2300 h (9.4 nmol/l; p = 0.03) (Figure 3b).

### Changes in insulin resistance

**Figure 4:**
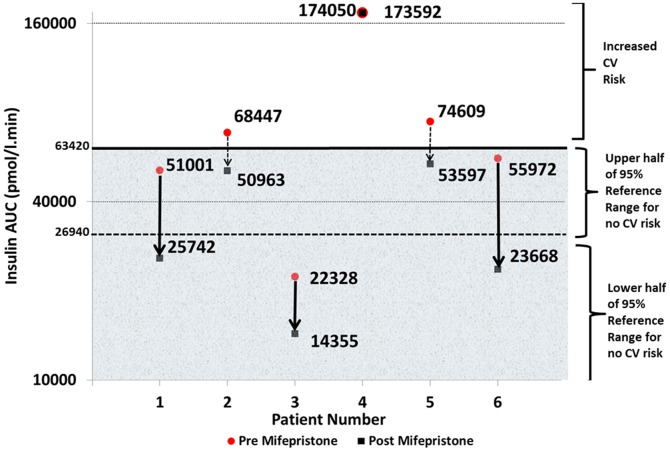
Graphs showing the changes in insulin AUC pre (red circle) and post (black square) mifepristone. The upper white area represents insulin AUC levels associated with increased risk for cardiovascular (CV) events. The grey shaded area represents the insulin AUC upper and lower 95% reference range (not associated with cardiovascular events). Mean: 26940 pmol/l.min; 2SD: 63420 pmol/l.min. Subjects 2 and 5 show a clinically significant improvement in their insulin AUC post mifepristone (dotted arrow). Subjects 1 and 6 both show a reduction in insulin AUC from the upper to the lower 95% reference range (solid arrow). Patient 4 remains at CV risk post mifepristone, indicating no clinical benefit. The insulin AUC is plotted on the y axis as a logarithmic scale to base 4.

As a group there were significant reductions in IR (lnHOMA-IR) and improvements in sensitivity (lnMatsuda index) compared to baseline following mifepristone treatment (Table 2). Five out of six individuals showed a reduction in insulin AUC (Figure 4). In subjects 2 and 5 there was a clinically significant reduction in insulin AUC from above the reference range into the upper half of the normal reference range. In subjects 1 and 6, insulin AUC fell by approximately 50% from the upper end of the reference range to the lower half of the reference range (<26940 pmol/l.min): patient 1: 51001 pmol/l.min to 25742 pmol/l.min; patient 6: 55972 pmol/l/min to 23668 pmol/l.min. In subject 3 insulin AUC fell, but this was within the lower half of the reference range at baseline. Subject 4 was highly insulin resistant and did not show any improvement in IR.

**Table 2:** Baseline and 4 week insulin sensitivity and glucose tolerance data after the use of mifepristone in 6 patients.

Variable (means ±95% CI)	Baseline	4 weeks	p value
Fasting glucose (mmol/l)	5.4 (4.6–6.2)	5.6 (5.0–6.1)	0.34
2-hour glucose (mmol/l)	10.2 (8.0–12.5)	11.4 (8.7–14.1)	0.21
AUC glucose (mmol/l.min)	1176 (1013–1339)	1187 (988–1384)	0.60
Geom Fasting insulin (pmol/l))	76.4 (39.9–146.4)	53.3 (25.8–109.9)	0.03
Geom 2-hour insulin (pmol/l)	628.5 (258.4–1528.3)	420.5 (159.4–1109.5)	0.20
Geom AUC insulin (pmol/l.min)	61973 (30889–124337)	40083 (16020–100289)	0.03
lnHOMA-IR	1.0 (0.2–1.7)	0.6 (−0.1–1.4)	0.03
lnHOMA-%β	4.8 (4.2–5.4)	4.3 (3.7–5.0)	0.03
lnMatsuda	1.2 (0.4–1.9)	1.6 (0.7–2.4)	0.03

Repeated measures effect analysed by non-parametric Wilcoxon's signed rank test.

AUC: Area under the curve HOMA - IR: Homeostatic Model Assessment of Insulin Resistance; HOMA - %β: Homeostatic Model Assessment of Beta Cell function.

### Changes in other metabolic markers

Subjects 2 and 5 improved their mean 24-hour BP from 143/75 to 135/67 and from 138/81 to 130/81, respectively. As a group there were no significant changes in resting or ambulatory blood pressure, serum osteocalcin and urine NTX/creat. There were no significant differences in the full lipid profile but interestingly there was a decrease in HDL in Subjects 2 and 5 from 0.93 mmol/l to 0.72 mmol/l and 0.84 mmol/l to 0.6 mmol/l respectively.

### Safety analysis

Five subjects completed the study to week four. One (subject 2) developed clinical symptoms of lassitude and fatigue at end of week two whilst another developed similar symptoms at the end of week four (subject 3). Importantly, there was no hypotension, indicating the relative clinical safety of using mifepristone in this setting despite the vague symptoms in these individuals. Data collected for withdrawn subject after week 2 were considered suitable for analysis as a significant drug effect is achieved by this time [15]. Serum potassium levels for these subjects were to 3.2 and 3.1 mmol/l, respectively, at the second and fourth week. There was a significant reversible change in thyroid stimulating hormone (TSH) (1.45 vs 4.07 mU/l; p = 0.03).

## Discussion

We have shown that short-term use of mifepristone can reduce IR in subjects with adrenal incidentalomas and low-grade autonomous cortisol secretion. To our knowledge, use of mifepristone in subjects with adrenal incidentalomas has never been tested. These data support recent findings in a study in patients with overt Cushing's syndrome where an improvement in insulin AUC and HOMA-IR was shown in the combined diabetes mellitus/impaired glucose tolerance and hypertension groups [12].

As a group we showed a significant reduction in IR and five of our subjects had a decrease in insulin AUC greater than 7237 pmol/l.min, exceeding biological variation. In addition, to establish whether GR blockade might also be associated with cardiovascular benefit, we defined our cut-off for insulin AUC from data published from the Helsinki Policemen Study, which investigated the relationship between baseline IR and the risk for cardiovascular disease over 22 years [10]. In that study, on an OGTT a mean (±SD) insulin AUC of 26940±18240 pmol/l.min was found in those without a future cardiovascular event. We used, therefore, the mean insulin AUC (±2SD) as our 95% reference range. Using this method we identified two subjects (numbers 2, 5) who had IR above this level but in whom IR decreased into the normal range with GR antagonism, consistent with a likely clinical benefit. Two other subjects (numbers 1, 6) experienced a reduction of their insulin AUC to the lower half of the reference range, whilst subject 3 had an insulin AUC value at baseline in the lower half of the reference range that improved further. Conversely, subject 4 has insulin resistance, which despite GR blockade, was not reversed. It may be that in some cases insulin resistance is more ‘fixed’ and less dependent on cortisol. We chose insulin AUC on OGTT as our primary estimate of IR as this allowed direct comparison to the seminal Helsinki heart study data examining the long term effects of IR on cardiovascular risk.

In addition to insulin AUC we used other estimates of insulin sensitivity. The Matsuda index of whole body insulin sensitivity, representing a composite of hepatic and peripheral tissues, and which considers insulin sensitivity in the basal state and after the ingestion of a glucose load, correlates strongly with estimates of insulin sensitivity from the ‘gold standard’ euglycemic insulin clamp (r = 0.73; p<0.0001) [21]. Although HOMA, or even stronger, log-transformed HOMA indices, correlate well with euglycemic clamps, they measure fasting steady state glucose and insulin concentrations and assume that hepatic and peripheral insulin sensitivity are equivalent [20], [23]. Nonetheless all insulin sensitivity indices significantly improved. Our data give strong support to the notion that even mild cortisol excess may be deleterious on insulin sensitivity/resistance, and that short-term antagonism of GR may be one means of teasing out which subjects have potentially reversible IR.

There remains an open question as to whether individuals who have been identified with adrenal incidentaloma should undergo any intervention. A number of studies have been carried out in individuals with cortisol secreting incidentalomas who have been subjected to adrenalectomy [26], [27] and improvements have been noted in weight, blood pressure, lipid profiles, fibrinogen levels, and glycemic control [11], [28], [29], [30], [31]. There is, however, no consensus on the criteria for the diagnosis of low-grade cortisol excess in patients with adrenal incidentalomas [13], [27] creating a dilemma on whom should be referred to surgery. Recently, tests of the HPA axis associated with known metabolic consequences of hypercortisolemia in these individuals have been suggested [32], but benefit from adrenal surgery has been demonstrated in subjects without ‘subclinical hypercortisolemia’ [26]. Whilst one interpretation is that this argues against a direct role for cortisol in their phenotype [27], an alternative interpretation is that the definition of ‘sub clinical’ is not correct, with some of those undergoing surgery being labeled as ‘normal’ in fact having clinically significant excess cortisol. Here, we have used dexamethasone suppression tests with a serum cortisol cut-off of 50 nmol/l. Diagnosis was supported by a low mean baseline 0900 h ACTH of 1.9 pmol/l. Whilst in a general population this strategy runs the risk of including too many false positives, in an individual with adrenal incidentalomas there is a considerable *a priori* likelihood that there is *some* abnormality of cortisol secretion, and thus a positive screening raises the post-test probability of hypercortisolemia to approximately 99% [18], [33]. It was for these reasons that we specifically focused on what would often be regarded as truly mild cases, to ask the question as to whether we would see any metabolic changes by antagonizing the GR without a pre-conceived definition ‘subclinical Cushing's/hypercortisolemia’; other than a diagnostic strategy that is in keeping with Endocrine Society guidelines [18]. Although we did not measure plasma dexamethasone levels there does not appear to be an advantage to using the 8 mg DST [34], and thus we feel that it is unlikely that our patients tested false positive.

As a group no significant changes were detected in the other metabolic markers investigated. Interestingly the two patients with clinically significant decreases in insulin AUC (patients 2 and 5) showed improvements in average 24-hour ambulatory blood pressure. Meta-analysis of several large prospective studies has shown that a 5 to 6 mm Hg decrease in diastolic blood pressure is associated with a 38% reduction in risk for stroke and a 16% reduction in coronary heart disease events [35], whilst a 10 mmHg reduction in systolic blood pressure is associated with a reduction in risk of stroke by one third [36]. Cortisol-induced hypertension is mediated by several mechanisms including an increased vascular responsiveness to catecholamines [37], elevated circulating catecholamine levels [38], and potentially, through a mineralocorticoid effect, when cortisol occupies the mineralocorticoid receptor if in excessive amounts consequent to HPA axis activation. The latter effect could explain why we did not see a significant change in BP in the group. The decrease in HDL although on first glance may seem non beneficial, in patients on mifepristone this has previously been shown to be associated with an increased efflux capacity of serum HDL on a per particle basis, hence no proportional impairment in HDL function [39].

As expected with glucocorticoid receptor antagonism the HPA axis was rapidly activated with serum and salivary cortisol and plasma ACTH levels rising appropriately. Hence, monitoring of changes in serum or salivary cortisol can be used to confirm subject compliance and drug efficacy. Throughout the study as indicated by the 0900 h/2300 h salivary cortisol ratio the physiological circadian rhythm of cortisol was maintained but the amplitude widened with maximal effects of mifepristone occurring in the morning. Limitations of our pilot study include the open design, a small sample and two patients were withdrawn after 2 and 4 weeks of mifepristone treatment, respectively. All patients received 400 mg per day, in two divided doses of 200 mg. The two patients who were withdrawn and who developed some symptoms of adrenal insufficiency were lighter, and hence had the highest relative dose, followed by highest HPA axis activation. Importantly, however, there was no hypotension, indicating the relative clinical safety of using mifepristone in this setting despite the vague symptoms in these individuals. These data reinforce the dose-dependent effect of mifepristone, with a dose of around 5 mg/kg/day being most appropriate. The nature of our study precludes generalization without further studies being performed.

In summary, we have shown that a short period of GR antagonism for up to four weeks in individuals with mild cortisol excess resulted in significant improvements in insulin sensitivity, and potentially clinically significant improvements in two out of six. These effects can now be examined in randomized controlled studies to establish whether GR blockade or cortisol-lowering strategies, are a suitable means for the individualized stratification of individuals with adrenal incidentaloma to medical or surgical intervention, or observation.

## Supporting Information

Checklist S1 **TREND Checklist.**
(DOC)

Protocol S1 **Trial Protocol.**
(DOC)

## References

[1] Mansmann G, Lau J, Balk E, Rothberg M, Miyachi Y, et al (2004) The clinically inapparent adrenal mass: update in diagnosis and management. Endocr Rev 25: 309–340. 15082524 10.1210/er.2002-0031

[2] Young WF Jr (2007) Clinical practice. The incidentally discovered adrenal mass. N Engl J Med 356: 601–610. 17287480 10.1056/NEJMcp065470

[3] Terzolo M, Stigliano A, Chiodini I, Loli P, Furlani L, et al (2011) AME position statement on adrenal incidentaloma. Eur J Endocrinol 164: 851–870. 21471169 10.1530/EJE-10-1147

[4] Newell-Price J, Bertagna X, Grossman AB, Nieman LK (2006) Cushing's syndrome. Lancet 367: 1605–1617. 16698415 10.1016/S0140-6736(06)68699-6

[5] Friedman JE, Yun JS, Patel YM, McGrane MM, Hanson RW (1993) Glucocorticoids regulate the induction of phosphoenolpyruvate carboxykinase (GTP) gene transcription during diabetes. J Biol Chem 268: 12952–12957. 7685354

[6] Vander Kooi BT, Onuma H, Oeser JK, Svitek CA, Allen SR, et al (2005) The glucose-6-phosphatase catalytic subunit gene promoter contains both positive and negative glucocorticoid response elements. Mol Endocrinol 19: 3001–3022. 16037130 10.1210/me.2004-0497

[7] Davani B, Khan A, Hult M, Martensson E, Okret S, et al (2000) Type 1 11beta -hydroxysteroid dehydrogenase mediates glucocorticoid activation and insulin release in pancreatic islets. J Biol Chem 275: 34841–34844. 10973946 10.1074/jbc.C000600200

[8] Qi D, Rodrigues B (2007) Glucocorticoids produce whole body insulin resistance with changes in cardiac metabolism. Am J Physiol Endocrinol Metab 292: E654–667. 17077342 10.1152/ajpendo.00453.2006

[9] Nosadini R, Del Prato S, Tiengo A, Valerio A, Muggeo M, et al (1983) Insulin resistance in Cushing's syndrome. J Clin Endocrinol Metab 57: 529–536. 6348064 10.1210/jcem-57-3-529

[10] Pyorala M, Miettinen H, Halonen P, Laakso M, Pyorala K (2000) Insulin resistance syndrome predicts the risk of coronary heart disease and stroke in healthy middle-aged men: the 22-year follow-up results of the Helsinki Policemen Study. Arterioscler Thromb Vasc Biol 20: 538–544. 10669654 10.1161/01.atv.20.2.538

[11] Tauchmanova L, Rossi R, Biondi B, Pulcrano M, Nuzzo V, et al (2002) Patients with subclinical Cushing's syndrome due to adrenal adenoma have increased cardiovascular risk. J Clin Endocrinol Metab 87: 4872–4878. 12414841 10.1210/jc.2001-011766

[12] Fleseriu M, Biller BM, Findling JW, Molitch ME, Schteingart DE, et al (2012) Mifepristone, a Glucocorticoid Receptor Antagonist, Produces Clinical and Metabolic Benefits in Patients with Cushing's Syndrome. J Clin Endocrinol Metab 10.1210/jc.2011-3350 22466348

[13] Chiodini I (2011) Clinical review: Diagnosis and treatment of subclinical hypercortisolism. J Clin Endocrinol Metab 96: 1223–1236. 21367932 10.1210/jc.2010-2722

[14] Des Jarlais DC, Lyles C, Crepaz N (2004) Improving the reporting quality of nonrandomized evaluations of behavioral and public health interventions: the TREND statement. Am J Public Health 94: 361–366. 14998794 10.2105/ajph.94.3.361 PMC1448256

[15] Kawai S, Nieman LK, Brandon DD, Udelsman R, Loriaux DL, et al (1987) Pharmacokinetic properties of the antiglucocorticoid and antiprogesterone steroid RU 486 in man. J Pharmacol Exp Ther 241: 401–406. 3572801

[16] Heikinheimo O (1997) Clinical pharmacokinetics of mifepristone. Clin Pharmacokinet 33: 7–17. 9250420 10.2165/00003088-199733010-00002

[17] Newell-Price J, Trainer P, Besser M, Grossman A (1998) The diagnosis and differential diagnosis of Cushing's syndrome and pseudo-Cushing's states. Endocr Rev 19: 647–672. 9793762 10.1210/edrv.19.5.0346

[18] Nieman LK, Biller BM, Findling JW, Newell-Price J, Savage MO, et al (2008) The diagnosis of Cushing's syndrome: an Endocrine Society Clinical Practice Guideline. J Clin Endocrinol Metab 93: 1526–1540. 18334580 10.1210/jc.2008-0125 PMC2386281

[19] Gordon BA, Fraser SF, Bird SR, Benson AC (2011) Reproducibility of multiple repeated oral glucose tolerance tests. Diabetes Res Clin Pract 94: e78–82. 21945562 10.1016/j.diabres.2011.08.025

[20] Matthews DR, Hosker JP, Rudenski AS, Naylor BA, Treacher DF, et al (1985) Homeostasis model assessment: insulin resistance and beta-cell function from fasting plasma glucose and insulin concentrations in man. Diabetologia 28: 412–419. 3899825 10.1007/BF00280883

[21] Matsuda M, DeFronzo RA (1999) Insulin sensitivity indices obtained from oral glucose tolerance testing: comparison with the euglycemic insulin clamp. Diabetes Care 22: 1462–1470. 10480510 10.2337/diacare.22.9.1462

[22] Perogamvros I, Owen LJ, Newell-Price J, Ray DW, Trainer PJ, et al (2009) Simultaneous measurement of cortisol and cortisone in human saliva using liquid chromatography-tandem mass spectrometry: application in basal and stimulated conditions. J Chromatogr B Analyt Technol Biomed Life Sci 877: 3771–3775. 10.1016/j.jchromb.2009.09.014 19783236

[23] Muniyappa R, Lee S, Chen H, Quon MJ (2008) Current approaches for assessing insulin sensitivity and resistance in vivo: advantages, limitations, and appropriate usage. Am J Physiol Endocrinol Metab 294: E15–26. 17957034 10.1152/ajpendo.00645.2007

[24] Tsagarakis S, Roboti C, Kokkoris P, Vasiliou V, Alevizaki C, et al (1998) Elevated post-dexamethasone suppression cortisol concentrations correlate with hormonal alterations of the hypothalamo-pituitary adrenal axis in patients with adrenal incidentalomas. Clin Endocrinol (Oxf) 49: 165–171. 9828902 10.1046/j.1365-2265.1998.00509.x

[25] Di Dalmazi G, Vicennati V, Rinaldi E, Morselli-Labate AM, Giampalma E, et al (2012) Progressively increased patterns of subclinical cortisol hypersecretion in adrenal incidentalomas differently predict major metabolic and cardiovascular outcomes: a large cross-sectional study. Eur J Endocrinol 166: 669–677. 22267278 10.1530/EJE-11-1039

[26] Chiodini I, Morelli V, Salcuni AS, Eller-Vainicher C, Torlontano M, et al (2010) Beneficial metabolic effects of prompt surgical treatment in patients with an adrenal incidentaloma causing biochemical hypercortisolism. J Clin Endocrinol Metab 95: 2736–2745. 20375210 10.1210/jc.2009-2387

[27] Stewart PM (2010) Is subclinical Cushing's syndrome an entity or a statistical fallout from diagnostic testing? Consensus surrounding the diagnosis is required before optimal treatment can be defined. J Clin Endocrinol Metab 95: 2618–2620. 20525910 10.1210/jc.2010-0633

[28] Midorikawa S, Sanada H, Hashimoto S, Suzuki T, Watanabe T (2001) The improvement of insulin resistance in patients with adrenal incidentaloma by surgical resection. Clin Endocrinol (Oxf) 54: 797–804. 11422115 10.1046/j.1365-2265.2001.01274.x

[29] Rossi R, Tauchmanova L, Luciano A, Di Martino M, Battista C, et al (2000) Subclinical Cushing's syndrome in patients with adrenal incidentaloma: clinical and biochemical features. J Clin Endocrinol Metab 85: 1440–1448. 10770179 10.1210/jcem.85.4.6515

[30] Terzolo M, Pia A, Ali A, Osella G, Reimondo G, et al (2002) Adrenal incidentaloma: a new cause of the metabolic syndrome? J Clin Endocrinol Metab 87: 998–1003. 11889151 10.1210/jcem.87.3.8277

[31] Toniato A, Merante-Boschin I, Opocher G, Pelizzo MR, Schiavi F, et al (2009) Surgical versus conservative management for subclinical Cushing syndrome in adrenal incidentalomas: a prospective randomized study. Ann Surg 249: 388–391. 19247023 10.1097/SLA.0b013e31819a47d2

[32] Morelli V, Masserini B, Salcuni AS, Eller-Vainicher C, Savoca C, et al (2010) Subclinical hypercortisolism: correlation between biochemical diagnostic criteria and clinical aspects. Clin Endocrinol (Oxf) 73: 161–166. 20184600 10.1111/j.1365-2265.2010.03794.x

[33] Elamin MB, Murad MH, Mullan R, Erickson D, Harris K, et al (2008) Accuracy of diagnostic tests for Cushing's syndrome: a systematic review and metaanalyses. J Clin Endocrinol Metab 93: 1553–1562. 18334594 10.1210/jc.2008-0139

[34] Reimondo G, Allasino B, Bovio S, Saba L, Ardito A, et al (2011) Pros and cons of dexamethasone suppression test for screening of subclinical Cushing's syndrome in patients with adrenal incidentalomas. J Endocrinol Invest 34: e1–5. 20634637 10.1007/BF03346701

[35] Hebert PR, Moser M, Mayer J, Glynn RJ, Hennekens CH (1993) Recent evidence on drug therapy of mild to moderate hypertension and decreased risk of coronary heart disease. Arch Intern Med 153: 578–581. 8439221

[36] Lawes CM, Bennett DA, Feigin VL, Rodgers A (2004) Blood pressure and stroke: an overview of published reviews. Stroke 35: 1024. 15053002

[37] Whitworth JA, Brown MA, Kelly JJ, Williamson PM (1995) Mechanisms of cortisol-induced hypertension in humans. Steroids 60: 76–80. 7792821 10.1016/0039-128x(94)00033-9

[38] Kumai T, Asoh K, Tateishi T, Tanaka M, Watanabe M, et al (2000) Involvement of tyrosine hydroxylase up regulation in dexamethasone-induced hypertension of rats. Life Sci 67: 1993–1999. 11072875 10.1016/s0024-3205(00)00787-6

[39] Page ST, Krauss RM, Gross C, Ishida B, Heinecke JW, et al (2012) Impact of mifepristone, a glucocorticoid/progesterone antagonist, on HDL cholesterol, HDL particle concentration, and HDL function. J Clin Endocrinol Metab 97: 1598–1605. 22399518 10.1210/jc.2011-2813 PMC3339893

