# Supplementary material for: Mifepristone Reduces Insulin Resistance in Patient Volunteers with Adrenal Incidentalomas That Secrete Low Levels of Cortisol: A Pilot Study
Source: PLoS One. 2013 Apr 5;8(4):e60984. doi: 10.1371/journal.pone.0060984 (PMC3618218; doi:10.1371/journal.pone.0060984)
Supplement: Protocol S1 — Trial Protocol. (DOC) [file pone.0060984.s002.doc]

**1. Project details**

**1.1 Details of Applicants**

**Investigator:**

Dr John Newell-Price MA PhD FRCP

Senior Lecturer and Consultant Endocrinologist

Academic Unit of Diabetes, Endocrinology & Metabolism

The University of Sheffield

Room OU142

O Floor

Royal Hallamshire Hospital

Glossop Road

Sheffield

S10 2JF, UK

Email: j.newellprice@sheffield.ac.uk

Tel: 00 44 (0) 114 226 1409

Secretary: Anne Lee

Tel. 0114 271 2162

Fax 0114 271 1863

e-mail [a.lee@sheffield.ac.uk](mailto:a.lee@sheffield.ac.uk)

**Co-investigator:**

Dr Miguel Debono, Academic Clinical Training Fellow

Address: S/A

Email: M.Debono@sheffield.ac.uk

**1.2 Sponsor: Sheffield Teaching Hospitals NHS Foundation Trust**

**1.3 Project Title:** A pilot study of the effect of glucocorticoid receptor antagonism in patients with Sub-Clinical Cushing’s syndrome.

**1.4 STH Project Reference Number:** 14971

**1.5 Protocol Version: 1.3, 27-09-08**

**1.6 Signatures**

**Chief Investigator**

**…………………………. ………………………… ……………………….**

**(Name) (Signature) (Date)**

**Sponsor Representative**

**………………………… ………………………….. ………………………..**

**(Name) (Signature) (Date)**

**1.7 EudraCT Number: 2007- 007604 - 15**

**Clinical Trials Authorisation (CTA) reference number: TBC**

**1.8 Trial Phase: Phase IIa**

**1.9 STH Programme affiliation:** Endocrinology and Reproduction

**2. Research Question.**

Does antagonism of the glucocorticoid receptor by use of mifepristone (RU486) improve blood pressure and indexes glycaemic control in patients with adrenal adenomas and sub-clinical Cushing’s syndrome?

This is a pilot study to assess if such antagonism has any effect in these patients. A larger study will then be planned based on the outcomes here.

**3. Aim of the Study**

To assess if short term (8 weeks) of antagonism of the glucocorticoid receptor with mifepristone improves metabolic parameters in this patient group as stated above. This is entirely original research. This will form the basis for a larger study. Ultimately it may lead to a diagnostic test that allows stratification of patients to adrenalectomy. This is a completely unmet need in clinical medicine, in an increasingly common clinical problem.

**4.Background**

**The need for study, scientific and clinical justification**

Cortisol excess is associated with increased mortality from cardiovascular disease. Cortisol excess in the general population is now recognised as being common, and is found frequently in patients with adrenal masses incidentally disclosed on CT scans, so called ‘adrenal incidentalomas’ ^1^. Compared to age, sex and BMI-matched controls, patients with these cortisol-secreting adrenal adenomas are at significantly increased cardiovascular risk with increases in hypertension, impaired glucose tolerance and diabetes, hyperlipoproteinaemia, and increased carotid intima-media thickness ^2-5^. Although the biochemical cortisol-excess is sufficient to causes these changes, it is insufficient to cause the clinical features typically associated with Cushing’s syndrome ^6,7^. It is for these reasons that the term ‘Sub-Clinical Cushing’s syndrome’ (SCS) is often applied to this condition.

Post mortem studies show a prevalence of adrenal adenomas of approximately 10% ^8^. Approximately 5% of all abdominal CT scans disclose an adrenal incidentaloma ^1,9^. The prevalence increases in an age-related fashion and they are found in 0.2% of abdominal CT scans in patients 20-29 years of age, this rising to approximately 10% in those over 70 years of age ^8,9^. Between 5-20% of these adrenal masses are associated with SCS ^10^. Thus, SCS is common in the general population (~1% or more of those >70y in hospitalized or health-screened populations), and contributes to overall cardiovascular morbidity and mortality. An ever expanding number of patients with adrenal masses are being found, due to the increasing use of CT in all areas of medicine: in the past decade the number of CT scans performed in the UK has doubled from 1.2 to 2.4 million per year (source Dept of Health, UK - form KH12). This huge increase means that the number of patients identified with SCS is set to rise still further.

The major problem is that management of SCS is *not* established. Approximately 90% of patients with SCS have hypertension, over 60% have impaired glucose tolerance or diabetes mellitus, and osteoporosis ^2,3,5,11-15^. In SCS there is the potential to permanently reduce these risks, and to improve bone health, by adrenalectomy. In terms of the potential benefit from treating these risk factors, meta-analysis of several large prospective studies has shown that a 5 to 6mm Hg decrease in diastolic blood pressure is associated with a 38% reduction in risk for stroke and a 16% reduction in CHD events ^16^, whilst a 10 mmHg reduction in systolic blood pressure is associated with a 31% reduction in risk of stroke ^17^. Moreover, impaired glucose tolerance is associated with a two-fold risk of cardiovascular death ^18^. Only a very limited number of individuals with SCS have been subjected to adrenalectomy, with the few reported forming parts of studies investigating the biochemical, cardiovascular and bone abnormalities of patients with adrenal incidentaloma. In those that have undergone this procedure improvements have been found in blood pressure (~10mmHg drop in systolic BP), lipid profiles, fibrinogen levels, and glycaemic control ^3-5,19,20^. However, the difficulty facing the clinician is in deciding whether adrenal surgery will be of benefit for a given patient with SCS, and the basis for selection for such permanent and invasive intervention is not established. On follow-up the majority of incidentalomas remain unchanged in size and malignant transformation is rare ^21^. In contrast to SCS the management of other causes of adrenal incidentaloma is not controversial, and the 4.2% that are phaechromocytomas and the 1.6% that are aldosteronomas ^22^ are usually considered for surgical excision. Surgery is also indicated if there is a significant increase in size demonstrated on CT scans repeated at intervals.

Given the current lack of knowledge in the management of SCS, we believe that short-term antagonism of cortisol by pharmacological glucocorticoid receptor blockade using mifepristone is a logical means to address these questions, and to test whether this improves clinically important risk factors in this common problem.

Specific questions to be addressed:

1. Does glucocorticoid receptor antagonism with mifepristone improve cardiovascular and metabolic derangements in patients with SCS?

2. Are there markers to predict or explain such a response?

Our approach will allow mechanistic study of the effects of such antagonism, without the inherent risks of surgery, and the opportunity to develop a clinical decision-making tool to inform the critical decision as to whether to proceed to adrenalectomy. We seek to perform an initial pilot study to assess the effects of this treatment and inform the design of a larger study.

Patients with adrenal adenomas with sufficient secretion of cortisol to cause clinical Cushing’s syndrome clearly benefit from adrenalectomy: uncontrolled Cushing’s syndrome has an SMR of 3.8-5.2 ^23,24^, this returning to normal with adequate control of hypercortisolaemia ^25^. Further insight into effects of the degree of excess glucocorticoids can be gained from important data studying the effects of exogenous glucocorticoids in the Scottish population, where even modest doses were associated with subsequent cardiovascular events, including stroke and myocardial infarction ^26^. Thus, the degree of hypercortisolaemia is important in determining the cardiovascular and metabolic events, but characterization of this compared to the outcome of any intervention is not established in SCS. To address this question in patients with SCS, one option would be to perform a study randomizing to adrenalectomy in order to assess the efficacy of lowering cortisol. This is, however, a highly interventional means of addressing the issue, and one that may not be to patients’ benefit. Furthermore, such a study could be more easily justified if short-term antagonism of cortisol was shown to be of benefit.

Three main tests are used to demonstrate excess cortisol secretion: urinary free cortisol, dexamethasone suppression tests, and midnight plasma or salivary cortisol ^6,7^. In SCS urinary free cortisol is usually within the normal range, as this is a relatively insensitive marker of hypercortisolaemia, whilst a post-dexamethasone serum value of >60nM (>1.9ug/dl) in patients with adrenal incidentalomas is associated with excess hypertension ^27^, as is an elevated midnight cortisol sample ^20^. Plasma ACTH levels are usually in the lower end of the normal range, reflecting low-grade partial hypothalamo-pituitary-adrenal axis suppression, as a consequence of the low-grade excess autonomous secretion of cortisol from the adrenal. In addition to these tests urinary steroid metabolite analysis by gas chromatography/mass spectrometry (GC/MS) is an invaluable tool allowing detailed analysis of the complete steroid output of an individual and, importantly, by analyzing substrate/product ratios, it facilitates the calculation of measures of steroidogenic enzyme activity. Decreased 5alpha-reductase activity is a specific feature of Cushing’s syndrome and distinguishes it from the polycystic ovary syndrome that is associated with increased 5alpha-reductase activity ^28,29^, though clinically both conditions may present with features of the metabolic syndrome (obesity, hypertension, impaired glucose tolerance). In addition urinary steroid GC/MS analysis allows the identification of decreased 11beta-HSD2 activity in severe overt Cushing’s syndrome due to the ectopic ACTH syndrome ^30^, a feature that is not apparent in SCS. GC/MS analysis will allow for a more detailed picture on the nature of glucocorticoid excess in adrenal incidentaloma than urinary free cortisol excretion alone. It will be established whether steroid excretion and metabolism in SCS differs from sex and age-matched patients with similar clinical features (obesity, and arterial hypertension). These controls will be drawn from ‘BUGS’, the Birmingham Urine Gas chromatography/mass Spectrometry database that contains data from more than 500 patients, including cohorts with polycystic ovarian syndrome, arterial hypertension, obesity and impaired glucose tolerance, in addition it contains data on normal controls of various ages with related oral glucose tolerance data (OGTT) data available in a significant proportion.

Mifepristone (11-[4-(Dimethylamino)phenyl]-17-hydroxy-17-[1-propynyl]-[11ß,17ß]-estra-4,9-dien-3-one), a derivative of the synthetic progestin norethindrone, is a potent competitive glucocorticoid and progesterone receptor antagonist, also known as RU 486 ^31^. Mifepristone has approximately three-fold higher affinity for the glucocorticoid receptor than dexamethasone, and 18-fold the affinity of cortisol ^32^. Mifepristone causes glucocorticoid antagonism by reducing translocation of the receptor to the nucleus and also by antagonising glucocorticoid-dependent transcriptional activity ^33^. Mifepristone is well absorbed and following oral ingestion has a bioavailability of more than 30%, and peak serum levels are reached within 1-2 hours, with a half-life of 24-48 hours ^34^. In man administration of mifepristone >200mg/day blocks central and peripheral glucocorticoid action, with resultant activation of the HPA axis: in normal volunteers 200 mg/day of mifepristone for eight days increased plasma, salivary, and urinary cortisol levels secondary to a slight rise in plasma ACTH, but without clinical or biological evidence of cortisol deprivation ^35^. Thus, these biochemical assessments allow confirmation of blockade of glucocorticoid action. On longer-term treatment, symptoms of adrenal insufficiency are possible, and highlight the need of clinical assessment, as the usual biochemical parameters of cortisol and ACTH levels cannot be reliably interpreted to assess this. Mifepristone-induced adrenal insufficiency can, however, be overcome: administration of dexamethasone 1mg is sufficient to overcome the effects of mifepristone 400mg ^32^. The efficacy of mifepristone has also been assessed in few patients with severe Cushing’s syndrome. Following the original description in 1985 ^36^ a total of 18 patients in the world literature in whom this drug has been used for this purpose have been described to 2007 ^37^. It has been found to be effective in patients with excess cortisol from adrenal tumours or the ectopic ACTH syndrome, and in reducing clinical parameters including hypertension, glucose homeostasis and psychosis. A study in severe ectopic ACTH syndrome is currently recruiting, with a starting dose of 600mg/day, titrating up to 20mg/kg/day. In addition to the anti-glucocorticoid effects, the anti-progestogenic effects appear at lower doses. The anti-progestogenic effects have also been exploited in the long-term treatment of patients with meningiomas, breast cancer (200-400mg/day) and myomas, with a good safety record (up to 14 years of treatment for meningiomas, mifepristone 200mg/day) ^38,39^. The anti-progestogenic effects of mifepristone have been also been shown to cause reversible endometrial hyperplasia after six months of therapy ^40^. Mifepristone is currently licensed at single doses of 600mg to induce medical termination of pregnancy, without evidence of adrenal insufficiency.

A variety of other compounds can be used to lower circulating cortisol by enzymic inhibition of the steroidogenesis in the adrenal, including metyrapone and ketoconazole, which are used routinely in the management of patients with Cushing’s syndrome. These compounds, however, also alter the synthesis of many other steroids, increasing androgenic and mineralocorticoid precursors, and are frequently associated with side effects.

*Hypothesis.* Based on the current literature and established effects of glucocorticoids on cardiovascular and metabolic risk, we hypothesise that mifepristone will improve blood pressure and glucose tolerance in patients with SCS, and that it may be used safely for this purpose.

*Why is the Study needed now?*

Because of the greatly increased use of CT an MR imaging in the UK and abroad, patients with identified SCS are common. Clinicians faced with this problem have no means of deciding how these patients should be best managed. It is for this reason that the recent NIH State of the Science Statement on the ‘Management of the Clinically Inapparent Adrenal Mass “Incidentaloma”, 2002’ ^41^, highlights the need for a prospective study to characterize sub-clinical hypercortisolism, including associated morbidities, and to establish the benefits of treatment. Although mifepristone has been in clinical use for many years it has never been used for this indication.

This pilot study will assess whether antagonism of low-grade hypercortisolaemia in patients with SCS improves the important established clinical endpoints of hypertension and glucose homeostasis. Furthermore, the design of the study includes assessment of parameters that will allow an understanding of the nature and degree of the mechanism of effect, and the length of time needed to see such an effect. There is clear potential patient benefit in establishing whether such an intervention improves these end points and other clinical parameters, and also in identifying markers that can be used to predict such an effect in clinical practice. The likely timelines for patient benefit would be rapid since the drug intervention is already available, facilitating translation into routine clinical practice.

This approach has not been considered before and is completely novel. All sources of evidence are summarized and referred to in the section above.

The study fits fully with the Endocrinology strategy for STH.

The study will be conducted in compliance with the protocol, GCP and applicable regulatory requirements.

**5. Plan of investigation**

**5.1 Methodology**

The full methodology and justification is given sections 5.2-5.10 below.

**5.2 Design**

This is an open labelled, pilot study over eight weeks at one centre: We feel that given the lack of widespread experience of using the anti-glucocorticoid actions of the drug in man in general, and the lack of data entirely for this indication, that the detailed nature of investigation is justified. As a compromise, the first 5 intervention visits are weekly, and the next two 2-weekly. A screening visit (week -4; visit 1) will also take place up to 4 weeks prior to the first intervention visit (week 0; Visit 2). A follow up visit will take place 72 hours after the last dose. We will recruit 6 individuals with SCS. The overall study design and assessments is shown below, including assessments made for secondary endpoints: Intervention - Mifepristone 200mg BD from week 0-8.

| **Week** | **-4** | **0** | **1** | **2** | **3** | **4** | **6** | **8** | **8** |
| --- | --- | --- | --- | --- | --- | --- | --- | --- | --- |
| Visit | 1 | 2 | 3 | 4 | 5 | 6 | 7 | 8 | 9 |
| Informed Consent | ✓ |  |  |  |  |  |  |  |  |
| Treatment (sc: screening; s: Treatment start; e: Treatment end; fu: Follow up 72 hours after last dose) | Nsc | Ys | Y | Y | Y | Y | Y | Ye | Nfu |
| Resting BP and clinical assessment | ✓ | ✓ | ✓ | ✓ | ✓ | ✓ | ✓ | ✓ | ✓ |
| 24-hour BP (during week of study) | ✓ |  |  |  |  | ✓ |  | ✓ |  |
| OGTT – 0,15,30,60,90,120 mins samples for insulin and glucose |  | ✓ |  |  |  | ✓ |  | ✓ |  |
| U+E | ✓ | ✓ | ✓ | ✓ | ✓ | ✓ | ✓ | ✓ |  |
| LFT | ✓ |  |  |  |  |  |  | ✓ |  |
| TSH | ✓ |  |  |  |  |  |  | ✓ |  |
| FBC | ✓ | ✓ |  |  |  | ✓ |  | ✓ |  |
| Fasting lipids |  | ✓ |  |  |  |  |  | ✓ |  |
| 0900h/2400h salivary cortisol | ✓ | ✓ | ✓ | ✓ | ✓ | ✓ | ✓ | ✓ |  |
| 0900h serum cortisol |  | ✓ | ✓ | ✓ | ✓ | ✓ | ✓ | ✓ |  |
| Plasma ACTH |  | ✓ | ✓ | ✓ | ✓ | ✓ | ✓ | ✓ |  |
| Bone turnover markers – formation, serum osteocalcin; resorption, urine NTX |  | ✓ |  |  |  |  |  | ✓ |  |
| Urinary steroid profile (during week of study) | ✓ |  |  |  |  |  |  | ✓ |  |
| Health-related quality of life questionnaires | ✓ |  |  |  |  | ✓ |  | ✓ |  |

***Inclusion criteria*** Patients will be eligible for inclusion if: they are males and over 18, or postmenopausal women; have an adrenal incidentaloma with benign characteristics diagnosed on CT or MRI; lack clinical features classically associated with Cushing’s syndrome; have evidence of excess cortisol as shown by lack of suppression of serum cortisol on 1mg over-night dexamethasone suppression or 2mg /day 48 hour low-dose dexamethasone suppression testing ^41^; stable antihypertensive and diabetic medication for two months prior to study entry.

***Exclusion criteria*** will include: evidence of local or systemic malignancy; overt Cushing’s syndrome; severe uncontrolled diabetes mellitus or hypertension; pregnancy; clinically significantly impaired cardiovascular function (e.g. stage IV cardiac failure); severe liver disease (liver enzymes ≥ 3 x the institutional upper limit of normal range); significantly impaired renal function (eGFR <30/min); uncontrolled severe active infection; treatment with approved or experimental steroidogenesis inhibitors, adrenolytic agents, within four weeks of admission; In women, known endometrial cancer, history of endometrial hyperplasia or vaginal bleeding of unknown cause; requirement for inhaled or systemic glucocorticoids for existing disease; impaired mental capacity or markedly abnormal psychiatric evaluation that precludes informed consent.

***Justification of period of study****:* The choice of eight weeks reflects the desire to allow sufficient period for the cardiovascular and metabolic effects of glucocorticoid antagonism to become manifest, and is extrapolated from the effects seen in the few patients with Cushing’s syndrome treated with mifepristone ^37^.

***Justification of dose and dose interval****:* The dose of 200mg twice daily has been chosen to provide partial blockade of glucocorticoid receptor activity. The interval is twice daily based on the known half-life of the drug and the wish to *partially* block glucocorticoid activity over the 24-hour period, without overt glucocorticoid deficiency, and to minimize the possibility of a rebound effect of increased cortisol exposure at the end of the dose interval, as may happen by using a single dose of mifepristone 400mg/24 hours.

***Drug administration and compliance****:* A check on the blister packs of the tablets issued and therapeutic drug monitoring will be performed at each visit to assess and encourage compliance. All existing medication will be left unaltered for the duration of the study. The first dose of the drug (Treatment start) will be given at Visit 2 (week 0) after baseline tests have been performed. The last dose (Treatment end) will be taken in the morning at Visit 8 (week 8). The drug will be administered 1 hour before food to maximise bioavailability as mifepristone is absorbed rapidly.

***Safety****:* Clinical assessment by BP, P, temperature, weight and questioning for fatigue, headache, anorexia, nausea, arthralgia, myalgia, and abdominal pain (potential glucocorticoid deficiency, a predictable effect of mifepristone based on its known action) will be made at each study visit. Clinical experience is, however, that even total adrenal insufficiency is well tolerated in the absence of physical or infective stressors. For safety purposes each patient will also be issued with written instructions which will tell them what to do if this side effect occurs and with a supply of two dexamethasone 1mg tablets (sufficient to overcome the effects of mifepristone 400mg by taking dexamethasone 1mg/day for 2 days) to be taken on the advice of the investigators if significant symptoms are reported. In such circumstances any study subject will be asked to attend the CRF either that day or the next working day, and data entered into the CRF accordingly, and the subject asked to stop the mifepristone and withdrawn. Other uncommon but documented side effects of mifepristone used at this dose and for this time period include: skin rash (maculopapular), increased eosinophil count, joint pain, hypokalaemia. When used for >3-6 months duration endometrial hyperplasia, uterine fibroma, and endometriosis are described.

**5.3 Statistical Analysis**

This protocol follows an open-label, non-randomised design. This study is exploratory in nature. All statistical analyses will be performed with descriptive and exploratory purposes and the results of statistical tests with confidence intervals, when given, will thus be considered an aid to evaluate the reliability of the observed result. The data will be summarised with respect to demographic and baseline characteristics, efficacy, observation and measurements, safety observations and measurements.

The primary outcomes analysed will be change in the resting and 24-hour ambulatory blood pressure, and the 2-hour glucose on OGTT. Secondary endpoints will be analysed in a similar fashion.

*Sample size*

This is a pilot study and no formal statistical analysis or power calculations will be performed. The number of patients has been selected to see if there is any trend in effect, and to assess the tolerability of the intervention. The information gathered will inform the design of a larger study, likely double-blind placebo controlled.

**5.4 Outcome measures**

***Primary end points*** will be the difference at 8 weeks, compared to baseline, in systolic blood pressure (BP) (resting and ambulatory) and 2-hour glucose tolerance during a 75g oral glucose tolerance test. BP will be measured in the sitting position, left arm taken twice, separated by 5 mins, after 10 mins resting (according to British Hypertension Society, UK [BHS] guidelines). 24-hour ambulatory blood pressure will be measured using standard BHS-approved monitors.

The primary endpoints have been chosen, as they are associated with important clinical outcomes. Baseline 24 hour ambulatory BP monitoring will be performed in the week starting treatment between the first visit (screening) and second visit (treatment start) and again at weeks 4 and 8. The daytime and nocturnal BP as determined by 24-hour ambulatory monitoring will be assessed independently and together to take account of the influence of the circadian rhythm in serum cortisol. The baseline glucose tolerance test will be performed on the first day of treatment (treatment start) before the first dose and again at weeks 4 and 8.

***Secondary end points*** will be compared to baseline: 1) the difference in BP and Glucose tolerance at 4 weeks; 2) homeostasis model assessment of insulin resistance (HOMA-IR) ^42^, and the insulin Sensitivity Index (ISI) ^43^ at 4 and 8 weeks, as calculated from oral glucose tolerance test: insulin and glucose at -15, 0, 30, 60 and 120 minutes, with oral glucose 75g at time 0 (except for those on insulin therapy, whose investigation will be limited to basal and 120 minute plasma glucose); 3) the difference in the mean plasma 0900h plasma ACTH, and salivary 0900 and 2400h cortisol values at 4 and 8 weeks; 4) the difference in fasting lipids at 8 weeks; 5) the difference in bone turnover markers at 8 weeks; 6) difference in urinary steroid profile at week 8; 7) the difference between depression, quality of life and fatigue questionnaires at 4 and 8 weeks. Tertiary endpoint will be any major cardiovascular adverse events at 8 weeks. Other biochemical safety parameters are summarized in the table above.

We considered other means of assessing glucose homeostasis, such as clamp studies, but felt that these would be too onerous for the participants and, importantly, not directly translatable to routine clinical practice. We accept that HOMA-IR is often quoted as being most appropriate for large epidemiological studies, but the correlation with euglycaemic clamp studies is high, and it has the advantage of simplicity and generalisability to clinical practice. Similarly the correlation between clamp studies and ISI is high.

The level of morning plasma ACTH and salivary nocturnal cortisol are anticipated to increase under the action of the study drug and give a biological marker for the effect of blockade. The repeated measurement will give greater confidence in the observation of drug activity. Salivary cortisol is stable at room temperature and thus can be collected by patients at home and brought or sent to the CRF. Similarly, assessment of the urinary steroid profile will be determined to assess the effects of mifepristone and the influence of increased circulating ACTH. Excess cortisol will suppress serum osteocalcin, a marker of bone formation, and thus the predicted effect of mifepristone would be an increase in this marker, and give insight as to the effect on bone health.

Clinically overt Cushing’s syndrome is associated with impairments in health-related subjective health status, that does not fully return to normal for many years after treatment ^44^. In SCS it is not established if quality of life is impaired. In view of this and the potential effects of altering excess cortisol on mood and general well-being, three validated questionnaires will be used to assess the effects of mifepristone therapy on health-related subjective health status. In addition to assessing any changes induced by mifepristone therapy the scores will be compared to sex and age–matched controls drawn from questionnaire-specific reference cohorts (available at the University of Sheffield), to establish if these parameters are impaired in SCS.

**5.5 Setting**

The Clinical Research Facility (CRF), Royal Hallamshire Hospital, Sheffield Teaching Hospitals Foundation Trust and University of Sheffield

**5.6 Participants**

Participants will be recruited from a group of patients who have attended the endocrine unit at the Sheffield Teaching Hospitals Foundation Trust and have already had clinical and biochemical evaluation for adrenal incidentalomas and been found to have evidence of SCS without the clinical phenotype of Cushing’s syndrome. Sheffield Teaching Hospitals Foundation Trust has one of the largest endocrine units in the UK, with six endocrinologists and a specialised laparoscopic adrenal surgeon, and receives direct referrals from a population catchment area of 3 million. All patients identified with an adrenal incidentaloma on CT/MRI are referred for assessment under a single protocol through this single point of referral, greatly facilitating recruitment.

**5.7 Recruitment**

We currently have 50 patients who fulfil the entry criteria, with a referral rate of 5-6 new patients per month. Therefore, there should be no issues over recruitment.

**5.8 Intervention**

Treatment will be in the form of an oral tablet of oral mifepristone twice-daily 200mg (0900h and 2100h) one hour before food, for eight weeks. Patients will attend the CRF at 0900h fasted at weekly intervals from weeks 0-4, and then again at weeks 6 and 8. A major advantage to this study design, compared to performing adrenalectomy, is that the treatment is immediately reversible, is not associated with the inherent risks of surgery, and it will inform design of a larger study, and tell whether an invasive approach by adrenal surgery is justified for study in this common patient group.

**5.9 Instruments**

Validated Health related quality of life scores (HRQOL) will be used as defined in section 5.4

A). *Depression* will be measured by Beck Depression Inventory ® (BDI®-II). The BDI-II takes approximately 10 minutes to complete. Each item has one numerical answer ranging from 0 (low depression) to 3 (maximum depression). Thus the total score ranges from 0 to 63.

B). *Quality of life* will be measured by the Short Form (SF-36), a 36-item health survey questionnaire to record general well-being during the previous 30 days and overall evaluation of health. Scores are expressed on a 0-100 scale, and higher scores are associated with a better quality of life.

C). *Fatigue* will be measured by Multidimensional Fatigue Index (MFI-20). This is a 5-point scale that comprises 20 statements to assess fatigue. Scores vary from 0-20, a high score indicating higher experienced fatigue.

**5.10 Project plan**

*Timelines***.** After regulatory and ethical approval it is anticipated that recruitment will be rapid and the study complete within 4-6 months. MHRA approval will be required. Mifepristone is already licensed for use in early pregnancy. MHRA regulatory approval was recently sought and granted to HRA Pharma for a multi-centre international study of the use of mifepristone in severe Cushing’s syndrome due to ectopic ACTH secretion. It is, therefore, anticipated that regulatory approval will be granted for this study.

Proposed timelines:

MHRA submission: in progress

Ethical submission: March 2008

Project Start date: November 2008

Last patient visit: May 2009

Database Lock: February 2009

Data analysis and write up March 2009

*Communication*

This is a pilot study and thus will not provide a definitive answer. Nevertheless, research findings will be disseminated by presentation at National and International meetings in both Endocrine and Cardiovascular areas, and by scientific publication, as and when appropriate. Engagement with the media will be facilitated by Newell-Price’s close working relationship with the UK Society for Endocrinology and US Endocrine Society, and their respective patient representation. Newell-Price is a trustee of UK-based patient charities.

Data Storage

Data will be collected and retained in accordance with the Data Protection Act 1998. The site file containing data will be available to the study staff and secured in a locked area on the CRF, RHH.

**6.0 Project management**

The investigator and independent physician will meet at least monthly to ensure that the project is progressing in a timely fashion (for ethical and governance issues see section 8).

**7.0 Expertise of the researcher and associated team**

The applicants involved have all the expertise necessary for such a trial. Newell-Price has a major interest in the assessment and treatment of glucocorticoid excess, a detailed knowledge of the biology, and experience of clinical trials in this area, and sees many patients with this condition as part of a dedicated diagnostic clinical protocol, and has many publications in the area of glucocorticoids. De Bono is an Academic Clinical Fellow and will gain invaluable experience in clinical research and be responsible for the day to day running of the study under the supervision of Newell-Price.

The clinical work will take place on the dedicated Clinical Research Facility (CRF) in Sheffield, which is jointly run by the University of Sheffield and The Sheffield Teaching Hospitals Foundation Trust. All of the facilities for performing the trial are available on the CRF including all necessary sample storage facilities.

**8.0 Ethical issues**

The study will be approved by the appropriate NHD Research Ethics Committees prior to commencement of the study. The study will be performed in accordance to the Declaration of Helsinki, ICH guidelines covering Good Clinical (research) Practice and the European Clinical Trials Directive and NHS Research Governance. A steering committee will be created according to GCP guidance. Dr Jonathan Webster (Sheffield) has already agreed to undertake this. Any adverse event will be flagged. An adverse event (AE) can be any unfavourable and unintended sign (including an abnormal laboratory finding), symptom, or disease temporally associated with the use of a medicinal product, whether or not considered related to the medicinal product.

If depression questionnaire highlights a level of depression requiring medical or psychiatric intervention necessary action will be taken by research team members.

Previous data suggests that mifepristone is well tolerated when taken for long periods of time (months to years). The main concern is the potential for inducing adrenal insufficiency.

*Safety and well-being of study participants*

Patients will be monitored for the non-specific symptoms and signs of relative adrenal insufficiency. It is unlikely that there will be any unanticipated side effects, but any potential adverse event will be recorded according to GCP guidelines and standard definitions.

#### *Interruption or discontinuation of treatment*

The term discontinuation refers to a patient’s non-compliance in the study. In this study, information about the discontinuation will be collected during the study. If in the opinion of the investigator, patients are considered to be treatment failures (i.e. require immediate alternative medical therapy) they will be discontinued from this study and the end of study evaluations will be completed.

Patients who discontinue the study prematurely must complete the final safety evaluations at the time of the discontinuation of their study. The study completion forms must be completed for all patients with an explanation of why the patient was withdrawn from the study, even if the patient refused to return for the final visits. Patients who discontinue prematurely due to their significant adverse events should continue to be followed until the resolution of the adverse event(s), and the relevant sections of the CRF should be completed as appropriate. Patients who discontinue due to clinically significant abnormalities in clinical laboratory results should continue to be evaluated until no abnormality results or is judged to be permanent.

It will be documented whether or not each patient completed the clinical study. If there are any patients participating in the study, and whose treatment or observations have been discontinued, the reason will be recorded. Reasons that a patient may discontinue participation are considered to constitute one of the following:

1. Adverse event(s)
2. Abnormal laboratory values
3. Abnormal test procedures
4. Unsatisfactory therapeutic effect
5. Subject’s condition no longer requires study treatment
6. Protocol violation
7. Subject withdrew consent
8. Lost to follow up
9. Administrative problems
10. Death

#### *Treatments and Blinding*

This is an open-label non-randomised study. Blinding is therefore not applicable.

*Concomitant therapy*

All medications taken during the four weeks prior to visit two will be recorded. Patients will be discouraged from taking any medication during the study that has not been taken at baseline. An exception is the medication that was required to treat an adverse event. Patients will not take any new prescription or over-the-counter medication during the study unless the Investigator has given their prior consent. Paracetamol is an acceptable concomitant medication, and if necessary, will be prescribed by a Physician. If any medication, is required, the name, strength, frequency of dosing, and reason for its use will be documented in the patient's Case Report Form. As mifepristone is metabolised by the CYP 3A4 enzyme system concomitant use of enzyme inhibitors or enzyme inducers may raise or reduce levels of mifepristone respectively. Mifepristone being an enzyme inhibitor of CYP 3A4 may lead to an increase in serum levels of drugs that are CYP 3A4 substrates. This factor should be taken into consideration if any of these drugs are taken.

*Contraception*

All male patients, including those who have been vasectomised, will be asked to use barrier contraception (e.g condom + spermicide) if they are sexually active with premenopausal women, throughout the whole trial and for a period of seven days after the last dose. This is to ensure there is no inadvertent transfer of mifepristone to a fetus in the semen.

Further, male patients and premenopausal partners should use two highly effective methods of contraception for three months from the last dose of mifepristone. This is to allow the passage of one spermatogenic cycle of 60-70 days after treatment has stopped before stopping contraceptive measures.

##### *Safety assessments*

##### Safety assessments will consist of monitoring and recording of all non-serious and serious adverse events and regular monitoring of blood chemistry, regular measurement of vital signs, and performing of physical examination and body weight estimation. Patients will be monitored for signs of adrenal insufficiency and will be educated on how to manage adrenal insufficiency and will carry a steroid card and will have a supply of 1mg oral dexamethasone to use in the event of symptoms (see above 2.3). A safety sheet assessment will be filled in at each visit by the investigator whilst on treatment.

Safety population – all patients who received at least one dose of Mifepristone will be considered for the safety evaluation.

### *Safety Evaluation* *- Adverse Drug Reactions*

Safety analysis will be performed on the safety population as defined above. The assessment of safety will be based mainly on the frequency of adverse events and on the number of laboratory values that fall outside the predetermined ranges. All adverse events will be listed. Adverse events will be summarised and presented with the number and percentages of patients having any adverse event and having an adverse event in each body system. Any other information collected (e.g. severity or relatedness to the study medication) will be listed as appropriate, and assessed by the investigator, co-investigator and steering committee. It is not anticipated that the use of mifepristone will be associated with severe adverse events. However, any such event will be reported to the STH Research Department within 24 hours, and any Suspected Unexpected Serious Adverse Reaction will be cascaded to the MHRA and Research Ethics Committee.

An adverse event (AE) can be any unfavourable and unintended sign (including an abnormal laboratory finding), symptom, or disease temporally associated with the use of a medicinal product, whether or not considered related to the medicinal product. All noxious and unintended responses to an IMP (i.e. where a causal relationship between an IMP and an adverse event is at least a reasonable possibility), related to any dose should be considered adverse drug reactions. For marketed medicinal products, a response to a drug which is noxious and unintended and which occurs at doses normally used in man for prophylaxis, diagnosis, or therapy of diseases or for modification of physiological function, is to be considered an adverse drug reaction. An unexpected adverse drug reaction is defined as an adverse reaction, the nature or severity of which is not consistent with the applicable product information (e.g. Investigator’s Brochure for an unapproved IMP).

### Serious Adverse Events (SAEs)

A serious adverse event (SAE) is defined as any untoward medical occurrence that at any dose either:-

• results in death

• is life threatening

• requires inpatient hospitalisation or prolongation of existing hospitalisation

• results in persistent or significant disability/incapacity (disability is defined as a

substantial disruption of a persons ability to conduct normal life functions)

• is a congenital anomaly/birth defect.

Important medical events that may not result in death, be life-threatening, or require hospitalisation may be considered a serious adverse drug experience when, based upon appropriate medical judgement, they may jeopardize the patient or patient and may require medical or surgical intervention to prevent one of the outcomes listed in this definition. Examples of such medical events include allergic bronchospasm requiring intensive treatment in an emergency room or at home, blood dyscrasias or convulsions that do not result in inpatient hospitalisation, or the development of drug dependency or drug abuse. Instances of death or congenital abnormality, if brought to the attention of the Investigator at any time after cessation of the study treatment and considered by the Investigator to be possibly related to the study treatment, will be reported to the sponsor.

Definition of Life Threatening:

An adverse event is life threatening if the patient was at immediate risk of death from the event as it occurred, ie. does not include a reaction that might have caused death if it had occurred in a more serious form. For instance, drug induced hepatitis that resolved without evidence of hepatic failure would not be considered life threatening even though drug induced hepatitis can be fatal.

Definition of Hospitalisation

Adverse events requiring hospitalisation should be considered serious. In general, hospitalisation signifies that the patient has been detained (usually involving an overnight stay) at the hospital or emergency ward for observation and/or treatment which would not have been appropriate at the CRF. When in doubt as to whether hospitalisation occurred or was necessary, the adverse event should be considered as serious. Hospitalisation for elective surgery or routine clinical procedures, which are not the result of an adverse event, need not be considered adverse events and should be recorded on a Clinical Assessment form and added to the Case Report Form. If anything untoward is reported during the procedure, this must be reported as an adverse event and either ‘serious’ or ‘non-serious’ attributed according to the usual criteria.

### SAE Reporting

STH R & D, the sponsor, will be responsible for coordinating the reporting of SAEs.

The Investigator will be responsible for completing an SAE report form and forward by fax to the sponsor immediately (within 24 hours) of becoming aware of an SAE.

The responsibilities of the sponsor include the following:-

• Prepare an adverse event reporting plan prior to the start of the study.

• Receive and review SAE report forms from the Investigator.

• Write case narratives and enter the case into the sponsor’s safety database

• Produce appropriate reports of all Suspected Unexpected Serious Adverse Reactions (SUSARs) and forward to the IEC, MHRA and the Principal Investigator.

Laboratory data will be summarised by presenting summary statistics of raw data and change from baseline values (means, medians, standard deviations, ranges) and the flagging of notable values in data listings.

Data from other tests will be listed. Notable values will be flagged. Any other information collected will be listed as appropriate.

#### *Efficacy*

Intention to treat population (ITT) - All patients who received at least one dose of Mifepristone and from whom at least one efficacy measurement is obtained after the study treatment start.

Protocol population (PP) - All patients who complete the study without any major violations of the protocol procedures.

##### *Efficacy evaluation*

The primary efficacy analysis can be run on both the ITT and PP populations in order to take into account the response of patients prematurely withdrawing from the study for any reason.

The primary efficacy evaluation is based on the primary efficacy parameters given. In cases of premature withdrawal from the study, at least one post-baseline measured efficacy parameter will be carried forward. The primary efficacy variable is then derived as the proportion of patients with successful treatment outcome after 8 weeks using the following criteria to define treatment outcome:

i) change in the resting and 24-hour ambulatory blood pressure.

ii) change in 2-hour glucose on OGTT.

*Safety of investigators* – There are no specific issues

*Participants rights to information and consent, confidentiality and privacy*

This will be in accordance to GCP guidelines. In terms of informed consent, patient recruitment will be at time after completion of usual investigation according to clinical practice. Since there is no specific time window needed for intervention, potential participants will have adequate time to consider their involvement and what it entails.

*Storage of data*

Patient data will be in a secure repository for 15 years.

*Ethnic and racial diversity*

This is a pilot study and thus the numbers are small. There is no evidence of altered adrenal function in different ethnic groups. In terms of information and consent if any potential study participant is not fully competent in the English language appropriate interpreters and translation of the information sheet and consent will be undertaken. Given the predominant Caucasian population of our catchment area it is likely that the participants will be Caucasian.

**9: Involvement of service users**

This is not anticipated from this pilot study

**10: Methods for disseminating research results**

This is pilot study and thus will not provide a definitive answer. Nevertheless, research findings will be disseminated by presentation at National and International meetings in both Endocrine and Cardiovascular areas, and by scientific publication, as and when appropriate. Engagement with the media will be facilitated by Newell-Price’s close working relationship with the UK Society for Endocrinology and US Endocrine Society, and their respective patient representation. Newell-Price is a trustee of UK-based patient charities.

**11: Strategy for taking the work forward if the research project is productive**

If pharmacological glucocorticoid receptor antagonism of patients with SCS in this study improves blood pressure and or glucose tolerance, this would pave the way for larger study using mifepristone (MRC application) and ultimately a formal intervention study with adrenalectomy in such patients. The magnitude of any effect observed would be used to inform the size of study. This would be planned by collaboration with the larger endocrine community in the UK and abroad to assess the potential for generalising the findings. A multi-centre study involving major endocrine centres with the expertise of laparoscopic adrenalectomy would be proposed, with the response to glucocorticoid antagonism prior to adrenalectomy to be correlated to overall outcome, and to specifically answer the question whether this results in an accurate way of stratifying patients to proceeding to this irreversible procedure. Our study design will also allow identification of a marker that best correlates with effective glucocorticoid antagonism and outcome. In certain patients at high operative risk pharmacological glucocorticoid receptor antagonism may be appropriate for longer-term therapy, and the effectiveness of this approach would need a study in larger populations.

**12: Intellectual Property arrangements**

A positive outcome from this study would have significant clinical implications. The next step would be to plan a formal intervention study including glucocorticoid antagonism followed by adrenalectomy. Biofusion PLC, the University of Sheffield’s partner in commercialisation, has already carried out an initial patent search (Derwent Innovations Index & Patent Hunter) and believes that mifepristone treatment of sub-clinical Cushing’s to be potentially novel and patentable – thus any commercial exploitation will accrue commercial benefit for the partners in this project, and ultimately UK PLC. It should be noted that Biofusion PLC and Medipex NHS Innovations have an excellent relationship and record of working together taking innovations to market and it is envisaged that they will work together on this project. It should also be noted that there are other companies that are in the pre-clinical stage of developing glucocorticoid receptor antagonists to which the results of this trial will be relevant. Thus, we believe that the investigators of this study will be in a good position to exploit a positive outcome of this study.

**13. Costing**

*Salary:* costs are covered by University of Sheffield for the investigator and co-investigator.

*Other recurrent expenses*: laboratory consumables, the use of the CRF, costs of assays, pharmacy costs, will be met by grants from the University of Sheffield.

**14. Funding source**

All costs will be covered by University of Sheffield accounts.

**15. References**

1. Young WF, Jr. Clinical practice. The incidentally discovered adrenal mass. *N Engl J Med* 2007;356(6):601-10.

2. Garrapa GG, Pantanetti P, Arnaldi G, Mantero F, Faloia E. Body composition and metabolic features in women with adrenal incidentaloma or Cushing's syndrome. *J Clin Endocrinol Metab* 2001;86(11):5301-6.

3. Rossi R, Tauchmanova L, Luciano A, et al. Subclinical Cushing's syndrome in patients with adrenal incidentaloma: clinical and biochemical features. *J Clin Endocrinol Metab* 2000;85(4):1440-8.

4. Tauchmanova L, Rossi R, Biondi B, et al. Patients with subclinical Cushing's syndrome due to adrenal adenoma have increased cardiovascular risk. *J Clin Endocrinol Metab* 2002;87(11):4872-8.

5. Terzolo M, Pia A, Ali A, et al. Adrenal incidentaloma: a new cause of the metabolic syndrome? *J Clin Endocrinol Metab* 2002;87(3):998-1003.

6. Newell-Price J, Trainer P, Besser M, Grossman A. The diagnosis and differential diagnosis of Cushing's syndrome and pseudo-Cushing's states. *Endocr Rev* 1998;19(5):647-72.

7. Newell-Price J, Bertagna X, Grossman AB, Nieman LK. Cushing's syndrome. *Lancet* 2006;367(9522):1605-17.

8. Kloos RT, Gross MD, Francis IR, Korobkin M, Shapiro B. Incidentally discovered adrenal masses. *Endocr Rev* 1995;16(4):460-84.

9. Mansmann G, Lau J, Balk E, Rothberg M, Miyachi Y, Bornstein SR. The clinically inapparent adrenal mass: update in diagnosis and management. *Endocr Rev* 2004;25(2):309-40.

10. Terzolo M, Reimondo G, Bovio S, Angeli A. Subclinical Cushing's syndrome. *Pituitary* 2004;7(4):217-23.

11. Tauchmanova L, Pivonello R, Di Somma C, et al. Bone demineralization and vertebral fractures in endogenous cortisol excess: role of disease etiology and gonadal status. *J Clin Endocrinol Metab* 2006;91(5):1779-84.

12. Francucci CM, Pantanetti P, Garrapa GG, Massi F, Arnaldi G, Mantero F. Bone metabolism and mass in women with Cushing's syndrome and adrenal incidentaloma. *Clin Endocrinol (Oxf)* 2002;57(5):587-93.

13. Chiodini I, Tauchmanova L, Torlontano M, et al. Bone involvement in eugonadal male patients with adrenal incidentaloma and subclinical hypercortisolism. *J Clin Endocrinol Metab* 2002;87(12):5491-4.

14. Osella G, Terzolo M, Borretta G, et al. Endocrine evaluation of incidentally discovered adrenal masses (incidentalomas). *J Clin Endocrinol Metab* 1994;79(6):1532-9.

15. Terzolo M, Osella G, Ali A, et al. Subclinical Cushing's syndrome in adrenal incidentaloma. *Clin Endocrinol (Oxf)* 1998;48(1):89-97.

16. Hebert PR, Moser M, Mayer J, Glynn RJ, Hennekens CH. Recent evidence on drug therapy of mild to moderate hypertension and decreased risk of coronary heart disease. *Arch Intern Med* 1993;153(5):578-81.

17. Lawes CM, Bennett DA, Feigin VL, Rodgers A. Blood pressure and stroke: an overview of published reviews. *Stroke* 2004;35(4):1024.

18. Glucose tolerance and mortality: comparison of WHO and American Diabetes Association diagnostic criteria. The DECODE study group. European Diabetes Epidemiology Group. Diabetes Epidemiology: Collaborative analysis Of Diagnostic criteria in Europe. *Lancet* 1999;354(9179):617-21.

19. Midorikawa S, Sanada H, Hashimoto S, Suzuki T, Watanabe T. The improvement of insulin resistance in patients with adrenal incidentaloma by surgical resection. *Clin Endocrinol (Oxf)* 2001;54(6):797-804.

20. Terzolo M, Bovio S, Pia A, et al. Midnight serum cortisol as a marker of increased cardiovascular risk in patients with a clinically inapparent adrenal adenoma. *Eur J Endocrinol* 2005;153(2):307-15.

21. Barzon L, Scaroni C, Sonino N, Fallo F, Paoletta A, Boscaro M. Risk factors and long-term follow-up of adrenal incidentalomas. *J Clin Endocrinol Metab* 1999;84(2):520-6.

22. Mantero F, Terzolo M, Arnaldi G, et al. A survey on adrenal incidentaloma in Italy. Study Group on Adrenal Tumors of the Italian Society of Endocrinology. *J Clin Endocrinol Metab* 2000;85(2):637-44.

23. Lindholm J, Juul S, Jorgensen JO, et al. Incidence and late prognosis of cushing's syndrome: a population-based study. *J Clin Endocrinol Metab* 2001;86(1):117-23.

24. Etxabe J, Vazquez JA. Morbidity and mortality in Cushing's disease: an epidemiological approach. *Clin Endocrinol (Oxf)* 1994;40(4):479-84.

25. Swearingen B, Biller BM, Barker FG, 2nd, et al. Long-term mortality after transsphenoidal surgery for Cushing disease. *Ann Intern Med* 1999;130(10):821-4.

26. Wei L, MacDonald TM, Walker BR. Taking glucocorticoids by prescription is associated with subsequent cardiovascular disease. *Ann Intern Med* 2004;141(10):764-70.

27. Valli N, Catargi B, Ronci N, et al. Biochemical screening for subclinical cortisol-secreting adenomas amongst adrenal incidentalomas. *Eur J Endocrinol* 2001;144(4):401-8.

28. Fassnacht M, Schlenz N, Schneider SB, Wudy SA, Allolio B, Arlt W. Beyond adrenal and ovarian androgen generation: Increased peripheral 5 alpha-reductase activity in women with polycystic ovary syndrome. *J Clin Endocrinol Metab* 2003;88(6):2760-6.

29. Stewart PM, Shackleton CH, Beastall GH, Edwards CR. 5 alpha-reductase activity in polycystic ovary syndrome. *Lancet* 1990;335(8687):431-3.

30. Stewart PM, Walker BR, Holder G, O'Halloran D, Shackleton CH. 11 beta-Hydroxysteroid dehydrogenase activity in Cushing's syndrome: explaining the mineralocorticoid excess state of the ectopic adrenocorticotropin syndrome. *J Clin Endocrinol Metab* 1995;80(12):3617-20.

31. Bertagna X, Bertagna C, Luton JP, Husson JM, Girard F. The new steroid analog RU 486 inhibits glucocorticoid action in man. *J Clin Endocrinol Metab* 1984;59(1):25-8.

32. Raux-Demay MC, Pierret T, Bouvier d'Yvoire M, Bertagna X, Girard F. Transient inhibition of RU 486 antiglucocorticoid action by dexamethasone. *J Clin Endocrinol Metab* 1990;70(1):230-3.

33. Beck CA, Estes PA, Bona BJ, Muro-Cacho CA, Nordeen SK, Edwards DP. The steroid antagonist RU486 exerts different effects on the glucocorticoid and progesterone receptors. *Endocrinology* 1993;133(2):728-40.

34. Heikinheimo O. Clinical pharmacokinetics of mifepristone. *Clin Pharmacokinet* 1997;33(1):7-17.

35. Bertagna X, Escourolle H, Pinquier JL, et al. Administration of RU 486 for 8 days in normal volunteers: antiglucocorticoid effect with no evidence of peripheral cortisol deprivation. *J Clin Endocrinol Metab* 1994;78(2):375-80.

36. Nieman LK, Chrousos GP, Kellner C, et al. Successful treatment of Cushing's syndrome with the glucocorticoid antagonist RU 486. *J Clin Endocrinol Metab* 1985;61(3):536-40.

37. Johanssen SaAB. Mifepristone (RU486) in Cushing's syndrome. *Eur J Endocrinol* in press.

38. Grunberg SM, Weiss MH, Spitz IM, et al. Treatment of unresectable meningiomas with the antiprogesterone agent mifepristone. *J Neurosurg* 1991;74(6):861-6.

39. Koide SS. Mifepristone. Auxiliary therapeutic use in cancer and related disorders. *J Reprod Med* 1998;43(7):551-60.

40. Newfield RS, Spitz IM, Isacson C, New MI. Long-term mifepristone (RU486) therapy resulting in massive benign endometrial hyperplasia. *Clin Endocrinol (Oxf)* 2001;54(3):399-404.

41. Grumbach MM, Biller BM, Braunstein GD, et al. Management of the clinically inapparent adrenal mass ("incidentaloma"). *Ann Intern Med* 2003;138(5):424-9.

42. Matthews DR, Hosker JP, Rudenski AS, Naylor BA, Treacher DF, Turner RC. Homeostasis model assessment: insulin resistance and beta-cell function from fasting plasma glucose and insulin concentrations in man. *Diabetologia* 1985;28(7):412-9.

43. Matsuda M, DeFronzo RA. Insulin sensitivity indices obtained from oral glucose tolerance testing: comparison with the euglycemic insulin clamp. *Diabetes Care* 1999;22(9):1462-70.

44. Lindsay JR, Nansel T, Baid S, Gumowski J, Nieman LK. Long-term impaired quality of life in Cushing's syndrome despite initial improvement after surgical remission. *J Clin Endocrinol Metab* 2006;91(2):447-53.

**16. Abstract**

Cortisol excess is associated with increased mortality from cardiovascular disease. In the general population this is now recognised as being common and is termed Sub-Clinical Cushing’s Syndrome, is found frequently in patients with adrenal masses incidentally disclosed on CT scans, and is associated with higher cardiovascular risk including hypertension and impaired glucose tolerance and diabetes. Pre-clinical and clinical data in other areas suggest that antagonism of the glucocorticoid receptor will improve these parameters, and guide selection for adrenal surgery. We propose an open-label pilot study to investigate the effects of the glucocorticoid receptor antagonist mifepristone in this condition. Mifepristone is currently licensed for obstetric practice. Outcome measures have been chosen that can predict clinical benefit, and that will allow an understanding of the nature and degree of the mechanism of effect. We propose to use oral mifepristone (200mg twice daily for 8 weeks), in one centre (Sheffield) to assess its effect in these patients. Primary endpoints will be resting and 24 hour ambulatory systolic blood pressure (BP) and 2-hour glucose on oral glucose tolerance testing at 8 weeks. Secondary end points at 4 and 8 weeks will be BP and glucose tolerance, insulin resistance and sensitivity, 0900h plasma ACTH/cortisol and salivary 0900/2400h cortisol values, and health related quality of life; secondary end points at 8 weeks will be fasting lipids, markers of bone turnover at 8 weeks, and urinary steroid profile (as determined by gas chromatography/mass spectrometry). A positive result of this study would be easy to translate into a larger clinical trial using mifepristone and also one including selection for laparoscopic adrenalectomy as a permanent therapy.

Health /Wealth

Cortisol excess in the general population is being increasingly recognised in patients with adrenal masses incidentally disclosed on CT scans (5% of all abdominal CT scans). Compared to age, sex and BMI-matched controls, patients with these cortisol-secreting adrenal adenomas are at significantly increased cardiovascular risk including hypertension and impaired glucose tolerance, especially in the aging population. There is no current standard of therapy for this condition. A successful outcome of this study would aid identification of those likely to benefit invasive intervention by surgery in this increasingly common problem.

**17. CV**

See appended CV

**18. Statistical advice**

This is not relevant for this pilot study since it is exploratory in nature. A larger and appropriately powered study with a placebo-controlled design will be planned pending the outcome of this study.

**19. Support Departments**

Helen Bowler, Clinical Trials Manager, Pharmacy, RHH

Martin Loxley, Site Laboratory Manager, Department of Clinical Chemistry, Royal Hallamshire Hospital
